# Obesity, non-alcoholic fatty liver disease and hepatocellular carcinoma: current status and therapeutic targets

**DOI:** 10.3389/fendo.2023.1148934

**Published:** 2023-06-08

**Authors:** Yinshuang Chen, Weipeng Wang, Maria P. Morgan, Tracy Robson, Stephanie Annett

**Affiliations:** ^1^School of Pharmacy and Biomolecular Sciences, Royal College of Surgeons in Ireland, University of Medicine and Health Sciences, Dublin, Ireland; ^2^College of Pharmaceutical Sciences, Soochow University, Suzhou, China

**Keywords:** hepatocellular carcinoma (HCC), obesity, nonalcoholic fatty liver disease (NAFLD), nonalcoholic steatohepatitis (NASH), therapeutic targets, animal models, metabolic dysfunction-associated fatty liver disease (MAFLD)

## Abstract

Obesity is a global epidemic and overwhelming evidence indicates that it is a risk factor for numerous cancers, including hepatocellular carcinoma (HCC), the third leading cause of cancer-related deaths worldwide. Obesity-associated hepatic tumorigenesis develops from nonalcoholic fatty liver disease (NAFLD), progressing to nonalcoholic steatohepatitis (NASH), cirrhosis and ultimately to HCC. The rising incidence of obesity is resulting in an increased prevalence of NAFLD and NASH, and subsequently HCC. Obesity represents an increasingly important underlying etiology of HCC, in particular as the other leading causes of HCC such as hepatitis infection, are declining due to effective treatments and vaccines. In this review, we provide a comprehensive overview of the molecular mechanisms and cellular signaling pathways involved in the pathogenesis of obesity-associated HCC. We summarize the preclinical experimental animal models available to study the features of NAFLD/NASH/HCC, and the non-invasive methods to diagnose NAFLD, NASH and early-stage HCC. Finally, since HCC is an aggressive tumor with a 5-year survival of less than 20%, we will also discuss novel therapeutic targets for obesity-associated HCC and ongoing clinical trials.

## 1 Introduction

Liver cancer is the sixth most frequently diagnosed cancer worldwide and has the third highest fatality rate of all cancers, with a 5-year survival of less than 20% (1, 2). The incidence of liver cancer is rising continuously and globally its mortality is expected to increase by 41% by 2040 (3, 4). Hepatocellular carcinoma (HCC), the most common form of liver cancer, accounts for over 90% of the cases (5). The occurrence of HCC is attributed to hepatitis B (HBV), hepatitis C (HCV), alcohol abuse, aflatoxin B1, iron accumulation, obesity and diabetes mellitus (6). Over the past decade, HBV and HCV infection are the primary risk factors for HCC, constituting 80% of HCC cases globally (7, 8). With the widespread availability of effective vaccination and antiviral therapies for HBV and HCV infection, the rates of viral-associated HCC are expected to decline in the coming years (9, 10).

Given the worldwide obesity pandemic, a growing amount of evidence suggests that obesity and the accompanying development of non-alcoholic fatty liver disease (NAFLD) and its aggressive form non-alcoholic steatohepatitis (NASH) are becoming the leading contributing factors to the rising incidence of HCC (11–13). Notably, efforts are underway to rename NAFLD as metabolic dysfunction-associated fatty liver disease (MAFLD), which emphasizes the role of the metabolic syndrome, obesity and Type 2 diabetes mellitus (T2DM) in contributing to the burden of liver disease (14). Obesity is a major driver of NAFLD and NASH, around 50% of NAFLD patients and 80% of NASH patients present with obesity (15). Notably, obesity itself is an independent risk factor for the onset and development of HCC. Obesity is associated with a 2-3 fold increased risk of HCC (16), and obese individuals exhibit an approximately 4-fold increase in HCC-related mortality and 2-fold increase in life-threatening complications following surgical cancer treatments (17–19). Obesity usually causes a diversity of complications, including cardiovascular diseases, insulin resistance (IR), T2DM, hypertension and hyperlipidemia (20–22). Indeed, IR and T2DM are also independent risk factors for chronic liver disease and HCC (23). The incidence of HCC among those with T2DM increased by 2 to 3-fold in different cohort studies (24, 25). In the context of HBV or HCV infection, the strong synergy between obesity and diabetes conveys more than a 100-fold HCC risk (26).

**Figure 1:**
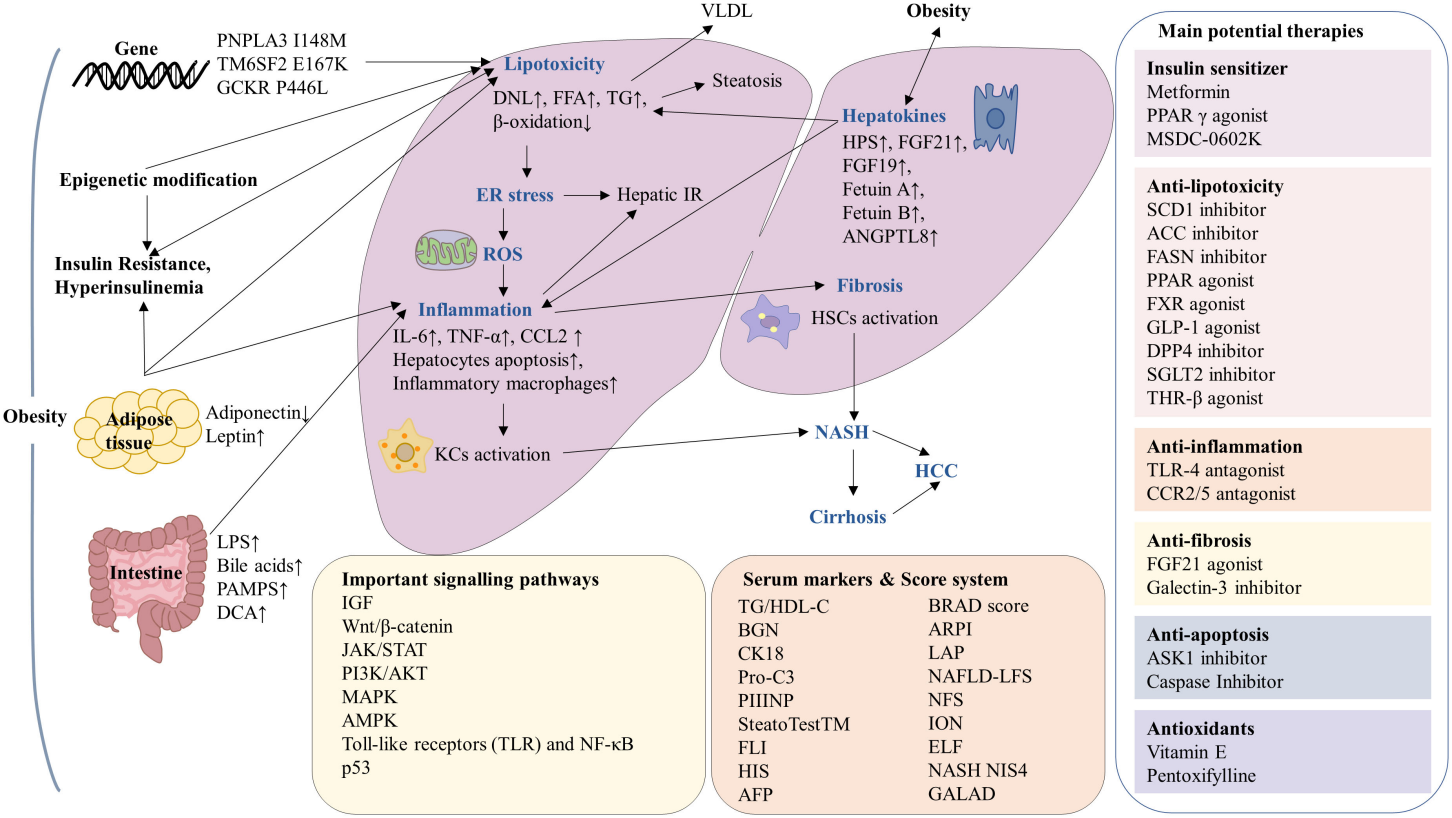
Summary of the mechanisms and serum markers of obesity-associated hepatocellular carcinoma (HCC) and current potential therapies. In the context of obesity, there is an increased risk of insulin resistant, gene variation, adipose tissue disfunction, epigenetic modification and intestinal microbiota imbalance, leading to lipotoxicity, endoplasmic reticulum (ER) stress, reactive oxygen species (ROS), inflammation and fibrosis, which ultimately progression to HCC. The molecular mechanisms involved in obesity-associated HCC including IGF, Wnt/β-catenin, JAK/STAT, PI3K/AKT, MAPK, AMPK, TLR, NF-κB and p53 signaling pathways. The serum markers and score system have been used in the diagnosis of NAFLD and NASH. Current potential therapies for obesity-associated HCC can be classified as insulin sensitizers, antioxidants as well as drugs against lipotoxicity, inflammation, fibrosis and apoptosis.

In principle, patients with HCC are stratified and allocated to treatment based on tumor stage, liver function and performance status (27). Resection, transplantation and local ablation are the first choices for patients with early-stage HCC tumors, while transarterial chemoembolization (TACE) is the first-line treatment for patients at intermediate stage and those with advanced stages will first receive systemic therapies (28). However, on average, patients are older and are more frequently diagnosed at advanced stages (29). Currently, systemic therapies including immune checkpoint inhibitors (ICIs), tyrosine kinase inhibitors (TKIs) and monoclonal antibodies are now improving the prognosis of HCC patients (28). However, ICIs may not be as effective for NASH-HCC as they are more appropriate for viral HCC. International clinical practice guidelines for HCC do not consider etiology, as there is insufficient data to draw specific conclusions or recommend etiology-specific treatment for patients with HCC (29). Furthermore, although the growing prevalence of obesity-associated HCC and vast studies are progressing, currently, there are no FDA-approved drugs and treatments for NASH yet. The therapeutic options for obesity-associated HCC are an unmet clinical need. A better understanding of obesity-associated HCC will help to establish more effective treatment strategies. Herein, this review discusses the underlying pathological mechanisms and signaling pathways of obesity leading to HCC. In addition, we summarize the novel potential therapeutic targets and ongoing clinical trials in HCC patients with obesity (**Figure 1**).

## 2 Pathophysiological mechanisms of obesity-associated HCC

### 2.1 Insulin resistance and hyperinsulinemia

Insulin resistance (IR) and subsequent hyperinsulinemia are major pathological consequences of obesity, which significantly contribute to the development of NAFLD, NASH and HCC (30, 31). Insulin is a key regulator of glucose metabolism and an increase in hepatic IR impairs glucose homeostasis by enhancing hepatic gluconeogenesis and glycogenolysis, leading to glucose intolerance (32, 33). Glucotoxicity is associated with elevated glucose levels and further contributes to IR (34). Both IR and hyperinsulinemia increase the serum level of insulin-like growth factor 1 (IGF-1) and the biological activity of IGF-1. IR and the binding of IGF-1 to insulin-like growth factor 1 receptor (IGF-1R) will trigger their downstream cellular pathways, such as phosphatidylinositol-3 kinase (PI3K), protein kinase B (AKT) and mitogen-activated protein kinase (MAPK), which induce HCC cells to proliferate and inhibit apoptosis, ultimately promoting the tumorigenesis of HCC (35, 36).

IR leads to a diverse range of metabolic and molecular effects including inflammation, endoplasmic reticulum (ER) stress and oxidative stress resulting in DNA damage which together contribute to hepatic cell injury and ultimately carcinogenesis in NASH (33, 37). Excessive lipid accumulation in liver is an important consequence of IR and the imbalanced energy metabolism leads to hepatic lipotoxicity and an increased release of free fatty acids (FFAs) in the serum and liver (38–40), with the deposition of large amounts of triglyceride (TG) in the liver which accelerates hepatocyte degeneration, fatty liver disease and fibrosis (41, 42). In hepatocytes, IR also causes steatosis through alterations in lipoprotein metabolism (33).

### 2.2 Lipid accumulation and lipotoxicity

The “lipid-rich condition” is highly characteristic of obesity-associated HCC and the deregulated hepatic lipid metabolism has been considered a driving force of HCC (43, 44). Hepatic lipid accumulation results from excessive lipid influx or impaired lipid export. Lipid accumulation includes four separate mechanisms 1) increased hepatic uptake of circulating fatty acids, 2), increased hepatic *de novo* lipogenesis (DNL), 3) decreased hepatic β-oxidation and 4) decreased hepatic lipid export. In obese individuals, the elevations of plasma FFA derived from adipose tissue depots and hepatic DNL promotes hepatic lipid accumulation, while hepatic β-oxidation and lipid secretion in very low-density lipoproteins (VLDL) decrease hepatic lipid content (45). Ectopic lipid accumulation in the liver is directly related to hepatic lipotoxicity, leading to exacerbation of steatosis and HCC development (46, 47).

Lipotoxicity is generally defined as the dysregulation of lipid environment and/or intracellular lipid composition, leading to an increased concentration of harmful lipids, impairing cellular homeostasis and disrupting tissue function (48, 49). Lipotoxicity ultimately leads to cell injury and chronic inflammation, followed by progression from NAFLD to NASH (50). Well-documented evidence indicates that the risk for lipotoxicity is also conveyed by FFAs rather than TG (51), suggesting that several underlying mechanisms that contribute to hepatocarcinogenesis. Lipotoxicity is a consequence of aberrant lipid metabolism. Hepatic metabolism of FFAs induces the formation of toxic metabolites, and they are responsible for inflammation, oxidative stress (OS) and liver parenchyma injury (51). The elevated FFA in hepatocytes promotes mitochondrial β-oxidation, which causes mitochondrial dysfunction and increased oxidative stress and leading to steatosis (52). FFAs have an additional function as signaling molecules, an energy source and structural components of the cell membrane, all of which are essential for cancer cell proliferation (43). FFAs are able to interfere with cellular signaling mechanisms and regulate gene transcription, activating various oncogenic pathways (53, 54). The overexposure of FFAs promote the expression of pro-inflammatory cytokines, impair insulin signaling and enhance apoptosis of hepatocyte in the context of ER and oxidative stress (51). In addition to direct cytotoxic effects, the accumulation of FFAs aggravates IR and hyperinsulinemia (55), which leads to further hepatic lipid accumulation (56), promotes inflammation (57) and increases carcinogenic fibrogenic responses (58) as well as mitogenic responses (56).

### 2.3 Adipose tissue and adipokines

Adipose tissue (AT) plays a major role in whole-body energy balance, as it responds rapidly and dynamically to changes in nutrient deprivation and excess through adipocyte hypertrophy and hyperplasia (59). AT expansion and progressive AT dysfunction is a key event in the development of obesity-associated HCC, due to the existence of adipose tissue-liver crosstalk (46, 60). AT remodeling is a continuous process that is pathologically accelerated in the obese state, and is characterized by a reduction in angiogenic remodeling, an overproduction of extracellular matrix (ECM), a heightened state of immune cell infiltration and subsequent pro-inflammatory response in obese individuals (59). AT is major locus of inflammation in obesity-related HCC (52, 61), and proinflammatory cytokine levels are markedly elevated in the AT of obese individuals. The accumulation of inflammatory cells, especially AT resident macrophages, in visceral AT, is a hallmark of AT dysfunction (62). The activation of inflammation and the recruitment of macrophages in visceral AT and subcutaneous AT of NAFLD patients correlates with the progression from simple steatosis to NASH and fibrosis (61, 63).

AT is an important energy storage organ and a key endocrine organ with active metabolism (64). The hormones (leptin, adiponectin), cytokines (tumor necrosis factor-alpha (TNF-α), interleukin-1beta (IL-1β), interleukin 6 (IL-6), and interleukin-8 (IL-8)), chemokines (Chemokine C-C motif ligand 2 (CCL2)), extracellular matrix proteins (matrix metallopeptidase 2 (MMP2) and matrix metallopeptidase 9 (MMP9)) and angiogenic proteins (vascular endothelial-derived growth factor (VEGF) are secreted from AT and collectively known as adipokines (65). Excess production of storage lipids leads to imbalanced adipokine secretion (adiponectin, leptin) (47, 66) that may profoundly affect not only the local AT itself but also the liver. The enhanced production of inflammatory chemokines and cytokines (TNF-α, IL-6) and reduced beneficial ones (adiponectin) contributes to acute and chronic inflammation as well as peripheral and hepatic IR (51). The expansion of AT, independent of other concomitant factors, deprives NAFLD patients of the anti-inflammatory and anti-fibrotic effects of adiponectin (65). In obese patients, increased leptin and decreased adiponectin level may lead to hepatic steatosis and activate inflammation and fibrosis (63).

### 2.4 Endoplasmic reticulum stress

Endoplasmic reticulum (ER) dysfunction is a common phenomenon in obesity-related HCC (67). ER stress is thought to drive adiposity by reducing energy expenditure (68) and emerging data suggest ER stress plays an important role in the progression of obesity, hepatic steatosis, NASH and HCC (69, 70).

An excessive influx of fatty acid leads to severe ER stress in obese states. In turn, ER enhances lipogenesis and hepatic steatosis (70). As a result, there is a positive feedback on ER stress and hepatic steatosis, which exacerbates liver damage (71). In addition, there is evidence that obesity-induced ER stress and inflammation in the liver can lead to hepatic IR (72). ER regulates protein synthesis and folding for various cellular processes. For instance, ER stress can induce hepatocyte apoptosis by activating C/EBP homologous protein and c-Jun amino-terminal kinases (JNK) signaling (48). ER stress initiates the unfolded protein response (UPR) to restore ER proteostasis, while UPR can cause inflammation and influence the development and aggressiveness of HCC (73). Oxidative stress is often accompanied by ER dysfunction. ER stress increases the production of reactive oxygen species (ROS) in hepatocytes, causing oxidative stress and subsequent genomic instability. In addition, ER and oxidative stress stimulate the sensitivity of hepatocytes to lipotoxic death, thereby releasing inflammatory mediators and inducing hepatic malignancy (74).

### 2.5 Oxidative stress

Oxidative stress is characterized by excessive levels of ROS. It is considered a tumor promoter by contributing to the initiation and progression of obesity-associated HCC (75, 76). The mitochondrial respiratory chain is the main source of hepatic oxidative stress derived from energy metabolism (77). In addition, other factors contributing to oxidative stress in obesity are: fatty acid accumulation, ER stress, chronic inflammation, abnormal postprandial ROS production, hyperleptinemia, tissue dysfunction and low antioxidant capacity (76).

Oxidative stress is not only a consequence but also a trigger of obesity and it plays a causative role in obesity development by stimulating white adipose deposition and increasing adipocyte proliferation (78). Hepatocyte exposure to excess ROS results in hepatocyte apoptosis and eventual cell death (79). Oxidative stress is closely linked to inflammation in obesity. Oxidative stress triggers the release of pro-inflammatory cytokines (TNF-α) and activates the inflammatory transcription factors (nuclear factor-kappa B (NF-κB) and activator protein-1 (AP-1)), thus leading to advanced fibrosis and cirrhosis, raising the risk of HCC (75, 76). Furthermore, the proinflammatory cytokines in turn enhance ROS production and cellular injury (75). Additionally, oxidative stress contributes to the release of pro-fibrogenic factors, which are involved in the initiation of fibrosis in HCC (79). Oxidative stress directly causes DNA alterations which leads to genomic instability and mutations in proto-oncogenes and tumor suppressor genes, thereby promoting tumor transformation (31).

### 2.6 Imbalance in intestinal microbiota

Intestinal microbiota plays an integral role in maintaining physiological, metabolic and enzymatic homeostasis (80). Sedentary lifestyle and high intake of a diet rich in saturated fat, sucrose and fructose have led to gut microbiota dysbiosis. Growing evidence has elucidated the association of gut microbiota dysbiosis with obesity, NAFLD and NASH (81–83), and it is a driving force in the progression of NAFLD and NAFLD-HCC through the gut-liver axis (84, 85).

Patients with NAFLD and NASH show significantly increased intestinal and detectable lipopolysaccharides (LPS) in portal blood. Alteration in intestinal microflora triggers inflammation, immune response, and immune cell infiltration of liver and AT (86). LPS is able to augment TNF-α production and activate the toll-like receptor 4 (TLR-4) pathway, thereby inducing a hepatic inflammatory response, leading to the progression of liver fibrosis and HCC (87, 88). In gut microbiota dysbiosis, pathogen-associated molecular patterns (PAMPs) are released. They are recognized by TLRs and potentiate innate immune responses (86). Bile acids are an important metabolite that links the gut microbiome with liver diseases (88). Dysregulated bile acid-microbiome crosstalk induces inflammation and HCC progression. Dysbiosis of the gut microbiome in obesity and NASH may induce the secretion of deoxycholic acid (DCA), a secondary bile acid that induces DNA damage. The high level of DCA in liver promotes the secretion of various inflammatory and tumor-promoting factors, thus further contributing to the development of HCC (89). Through the modulation of the gut-liver axis, the gut microbiota dysbiosis causes increased intestinal permeability, transfer of LPS, unrestricted transfer of microbial metabolites to the liver, immune activation and altered bile acid signaling, all of which contribute to liver inflammation, fibrosis, and eventually proceed to HCC (81, 90), thus further confirming the crucial role of the gut-liver axis in the pathogenesis of HCC.

### 2.7 Inflammation and Immune response

Obesity is characterized by a low-grade chronic inflammation which is a pivotal component for HCC development in the context of obesity (46). In obese individuals, the inflammation is attributed to the exacerbation of inflammatory cytokines deriving from extrahepatic sites (e.g. AT expansion and intestinal inflammation), or within the liver, the activation of Kupffer cells (KCs) and lipotoxicity of hepatocytes (51, 91).

The pathological cascade associated with inflammation leads to the activation of hepatic stellate cells (HSCs) and their fibrogenic differentiation, ultimately leading to liver fibrosis and cirrhosis (92). Chronic inflammation facilitates the massive release of proinflammatory cytokines (IL-6, TNF-α) (66). Through activity on multiple oncogenic molecular pathways (e.g. inhibitor of kappa kinase IKK/c-Jun amino-terminal kinases JNK, signal transducer and activator of transcription STAT and NF-κB pathways), high levels of cytokines trigger IR, oxidative stress, lipotoxicity, hepatocyte cell death, liver inflammation, fibrosis, and pathological angiogenesis, thus promoting the progression from simple obesity-related hepatic steatosis to HCC (26, 80, 93).

Obesity modulates intrahepatic immunity and induces a microenvironment of immune intolerance, which leads to the progression of HCC (94). In the setting of obesity, pro-inflammatory cytokines, lipotoxicity and intestinal flora affect the activation of innate and adaptive immunity by stimulating liver-resident macrophages, named KCs, and recruitment of inflammatory macrophages to the liver (94–96). Hepatocytes and KCs secrete chemokines, including CCL2, thereby increasing the liver macrophage pool through monocyte infiltration (97). The recruitment of these immune cells to the liver is an important step in the pathogenesis of NASH and HCC (98). Activation of innate immunity drives further hepatic infiltration and accumulation of inflammatory cells, thereby aggravating inflammation and damage to the liver, regulating the progression of liver fibrosis, angiogenesis and carcinogenesis (95). Intrahepatic activated CD8^+^ T cells and natural killer T (NKT) cells are increased in NASH (99). NKT cells are able to secrete TNF superfamily member 14 (TNFSF14), which increases FFA uptake in hepatocytes and induces steatosis. Through interactions with hepatocytes, CD8^+^ T cells and NKT cells cooperatively induce liver damage and steatosis (100). Obesity decreases the population of CD4^+^ T cells which play a critical role in NAFLD-HCC progression and loss of hepatic CD4^+^ T cells compromises immunotherapies, such as RNA vaccine (M30) and anti-OX40 antibody-mediated therapy against tumor cell growth in the liver (101, 102).

### 2.8 Autophagy

Autophagy is a lysosome-dependent catabolic process that contributes to hepatic homeostasis through its role in energy balance and cytoplasmic quality control, removing misfolded proteins, damaged organelles and lipid droplets (103). Autophagy shows beneficial or deleterious effects, depending on the cell type. Autophagy regulates the breakdown of lipid droplets and prevents liver injury in hepatocytes, exerts anti-inflammatory effects in macrophages, while autophagy has pro-fibrogenic properties in HSC (51, 84, 104).

Growing evidence suggests that autophagy is inactive during obesity and NAFLD. Obesity and its associated metabolic stress can interfere with the autophagic process, leading to the promotion of retention of damaged mitochondria, elevated oxidative stress and activation of DNA damage responses, accelerating obesity-related pathology in the liver, adipose and gut (105). This dysregulation of autophagy has been linked to many liver diseases, including HCC (84). In the obese state, the excess of TG and FFAs inhibit the initiation of autophagy through activation of mammalian target of rapamycin (mTOR) and suppression of unc-51 like autophagy activating kinase 1 (ULK1) activity (106). The decreased autophagic function contributes to hepatic oxidative stress, steatosis and other pathophysiology of HCC (107). Autophagy suppresses tumorigenesis by blocking cell damage or facilitating the removal of tumorigenic initiating cells, and thus, impairment of autophagy may be a causal factor in the development of HCC in advanced NASH. In addition, a change in autophagic activity plays a critical role in the development of immune response, insulin sensitivity, diabetes and other metabolic diseases, which promotes HCC development (107, 108). Collectively, all the above suggests autophagy may be a therapeutic target in obesity-associated HCC.

### 2.9 Hepatokines and metabolism

Accumulating evidence reveals that obesity accelerates the secretion of hepatokines from hepatocytes such as hepassocin (HPS), angiopoietin-like protein 8 (ANGPTL8), Fetuin-A and B and fibroblast growth factor 19 and 21 (FGF19/21) (109, 110). Hepatokines mainly act as liver-derived pro-inflammatory factors, playing an essential role in inducing liver steatosis and NASH to HCC by modulating the lipid metabolism progress, ROS production, inflammatory response and other oncogenic conditions (111, 112). In turn, hepatic steatosis and HCC induce the secretion of ectopic hepatokines and play an alternative role in the pathogenesis of obesity (96). In addition, the associated metabolic changes caused by hepatokines alter the secretion of other organokines that play a regulatory role in the pathogenesis of NASH (113).

Elevation of these hepatokines in plasma has been associated with HCC development or a poor prognosis in NAFLD-related HCC. For instance, HPS overexpression facilitates hepatic lipid accumulation and promotes inflammatory cytokines and lipogenic gene expression (114). A high concentration of Fetuin-A is associated with IR and enhances the release of pro-inflammatory cytokines, inducing a lipotoxic pro-inflammatory response (112). ANGPTL8 is highly expressed in liver and AT, its overexpression is positively correlated with hepatic steatosis, lipogenesis and tumor cell proliferation (114). Hepatokines may be considered biomarkers of ectopic fat accumulation in the liver and markers of the disease progression, some of them may be the target for the prevention and treatment of IR and HCC (113, 115).

### 2.10 Genetic factors

The obesogenic environment exposes a disease risk associated with genetic variants, including NAFLD, NASH and HCC. Genetic factors may be responsible for the individual susceptibility and clinical course of NAFLD. Multiple studies have emphasized that specific genetic variations predispose to NAFLD susceptibility and NAFLD-related HCC (31). Single nucleotide polymorphisms (SNPs) in genes, including human patatin-like phospholipase domain-containing 3 (*PNPLA3*), transmembrane 6 superfamily member 2 (*TM6SF2*), glucokinase regulator (*GCKR*), membrane-bound O-acyltransferase domain-containing 7 (*MBOAT7*), hydroxysteroid 17β-dehydrogenase 13 (*HSD17B13*), are associated with NASH development and they are associated with regulation of hepatic fat content, plasma liver enzyme levels and glucose metabolism (116, 117). For example, an SNP rs738409 C/G in *PNPLA3* results in an isoleucine to methionine substitution at residue 148, which is designated *PNPLA3* I148M. The effect of the *PNPLA3* I148M genetic variation is significant, with each allele having approximately a 2-fold increase in the odds of NAFLD and a 3-fold increase in the odds of NASH and HCC (118). An SNP in *TM6SF2* encoding a glutamate to lysine substitution at amino acid position 167 of TM6SF2 protein (E167K) is associated with increased DNL, reduced secretion of apolipoprotein B particles, promoting the development of NASH, advanced hepatic fibrosis and cirrhosis (118, 119). All SNPs in *TM6SF2, GCKR, MBOAT7* and *HSD17B13* are shown to be associated with *PNPLA3* I148M, affecting all stages of NAFLD, suggesting that these genetic variants have additive effects on the progression of NAFLD and NAFLD-related HCC (84). In addition, obesity interacts with *PNPLA3* I148M genetic variation to elevate liver fat content and NAFLD susceptibility, and to increase the risk of liver injury, liver fibrosis and HCC (120–122). *PNPLA3* I148M has a more severe effect on liver injury in people with obesity than in lean individuals. Obesity also amplifies the interaction of *PNPLA3* I148M with alanine transaminase ALT level and cirrhosis. Other studies also report the interactions of obesity with *TM6SF2* E167K and *GCKR* P446L (123, 124). Genetic variation combined with obesity, increased abdominal fat mass and excessive carbohydrates may confer a higher risk of developing HCC (125).

### 2.11 Epigenetic modification

Epigenetic regulation of gene expression via DNA methylation, histone modification and microRNAs (miRNAs) are all associated with NAFLD development (126). Epigenetic alterations occur when exposed to an obese or nutrient-rich environment (127). Excessive glucose, lipid and insulin-generated metabolites may disrupt the epigenetic balance, thereby altering transcriptional networks involved in redox homeostasis, peroxisome and mitochondria function, inflammation, insulin sensibility and lipid homeostasis, driving NAFLD development and NAFLD-associated HCC tumorigenesis (126, 128).

DNA methylation is the most reported epigenetic modification (129). Accumulating investigations show the key genes responsible for metabolic, lipid homeostasis, insulin signaling, DNA repair, remodeling of liver tissue and fibrosis progression are significantly and differentially methylated (126) (130). Dipeptidyl peptidase 4 (DPP4), an adipokine released by hepatocytes, is known to be upregulated in the liver of patients with obesity and NAFLD, while methylated *DPP4* is negatively correlated with the stages of hepatic steatosis and NASH (131). A previous study showed that hypermethylation of the peroxisome proliferator-activated receptor gamma (*PPARG*) promoter was associated with fibrosis severity in liver biopsies (132).

Histones undergo various modifications such as acetylation, phosphorylation, methylation, ubiquitination, SUMO-ization and ribosylation, of which acetylation has been extensively reported (133). Histone acetylation promoted by histone acetylase (HAT) activates gene transcription, while histone deacetylation catalyzed by histone deacetylase (HDAC) promotes gene silencing. It has been reported that altered expression and function of HAT and HDAC affect hepatic metabolism and cellular transformation in NAFLD (134). P3000, a member of the HAT family, is involved in regulating the transcription of the NF-κB pathway, glycolytic and lipogenic genes, and contributes to hepatic steatosis and NAFLD development (128, 133). One study found that inhibition of p300 improved MAFLD in mice, restored biochemical parameters and reduced the activity of genes involved in adipogenesis (135).

It is well documented that several miRNAs are considered to be critical mediators of metabolic diseases including obesity, T2DM, metabolic syndrome and MAFLD (136, 137). These miRNAs encompass miR-27b, miR-33, miR-34a, miR-103, miR-107, miR-122 and miR-223, which play an essential role in controlling the metabolism and homeostasis of insulin, glucose, cholesterol and lipid (128). miR-122 is a liver-rich and liver-specific miRNA with key roles in liver metabolism, cholesterol biosynthesis, fatty acid synthesis and oxidation (138). Systemic or specific deletion of miR-122 in the liver showed a significant decrease in total serum cholesterol (TC) and TG levels and a marked improvement in hepatic steatosis, suggesting that miR-122 is a crucial regulator of cholesterol and fatty acid metabolism in the liver and a potential therapeutic target for NAFLD (128, 138).

### 2.12 Fatty metamorphosis

Fatty metamorphosis is a prominent histologic characteristic of well-differentiated HCCs, and HCCs show various degrees of fatty metamorphosis (139, 140). Fatty metamorphosis can be classified as diffuse and focal forms. Diffuse metamorphosis is found throughout the cancer nodule, whereas focal metamorphosis localizes in part of the nodule. The frequency of fatty metamorphosis is highest in HCCs with a diameter of 11-15 mm, and the type of metamorphosis may transition from diffuse to focal (140). Some studies suggest that the possibility of HCC should be considered when focal fatty metamorphosis is found in the cirrhotic liver (139). Fatty metamorphosis is thought to be related to ischemia and metabolic disorder, including obesity, diabetes and hyperlipidemia (141). In hepatic fatty metamorphosis, triglycerides are deposited in the hepatocytes, effectively converting the cells into adipocytes (142). A study found that the severity of fatty metamorphosis is increased from normoglycaemic to diabetic obese patients (143).

## 3 Molecular mechanisms of obesity-associated HCC

### 3.1 IGF pathway

The IGF axis consists of three ligands (insulin, IGF-1 and IGF-2), three receptors (insulin receptor, IGF-1R and IGF-2R), substrates (insulin receptor substrate IRS) as well as ligand binding proteins (144). Dysregulation of IGF signaling plays a critical role in the pathogenesis and carcinogenic of HCC, particularly in obesity-associated HCC (31). Current evidence indicates that insulin and hyperinsulinemia promote the synthesis and biological activity of IGF-1 and IGF-2, which regulates the energy-dependent growth process (31, 36).

IGF-1 is mainly secreted by the liver, and it can act as an autocrine, paracrine or endocrine growth factor. IGF-1 has a higher affinity for IGF-1R, which is involved in the generation of preneoplastic lesions (145). The binding of IGF-1 and IGF-1R is able to regulate stem cell pluripotency and differentiation, triggering cell proliferation, organ development and tissue regeneration (33). In addition, imbalanced IGF-1/IGF-1R signaling stimulates HCC cell proliferation and inhibits apoptosis through activating MAPK pathway and c-JNK pathway. IGF-1 also promotes angiogenesis by increasing the production of VEGF (146). Plasma level of IGF-2 is increased in obese, T2DM patients, and cirrhosis as well as HCC (144). Similar to IGF-1, IGF-2 is also produced in the liver. During hepatocarcinogenesis, IGF-2 has a variety of oncogenic functions via binding to IGF1R, such as inhibiting apoptosis, promoting HCC cell proliferation and migration, and activating angiogenesis (147). IRS-1, the main substrate of IGF-1R activation, is a key component of IGF axis. Studies have demonstrated IRS-1 acts as a dominant oncogene and has a higher level in HCC (148). Hyperinsulinemia and elevated IGFR activates the phosphorylation of IRS-1, resulting in the activation of multiple cytokine pathways, including PI3K/AKT/mTOR and MAPK cascade, which modulate cell cycle and may potentially enhance tumor progression of HCC (35).

### 3.2 Wnt/β-catenin pathway

Wnt/β-catenin signaling is one of the most important pathways required for cell fate differentiation and overall maintenance of liver metabolism and homeostasis (149). Dysregulation of the Wnt/β-catenin pathway and its various components effects NAFLD progression, starting with early obesity, diabetes, NASH and progressing to fibrosis, cirrhosis and HCC. In turn, evidence suggests that Wnt activity is enhanced in liver cirrhosis, and it is frequently hyperactivated in HCC patients (149, 150). An aberrant activation of Wnt/β-catenin signaling is a hallmark of various hepatic pathologies, it plays a role in almost every aspect of liver biology (151).

β-Catenin, encoded by *CTNNB1*, is the core protein of the Wnt signaling cascade. Central to the pathway is the interaction of Wnt ligand with Frizzled/low-density lipoprotein receptor-related protein (LRP) co-receptor complex, β-catenin accumulates aberrantly in the nucleus, leading to the expression of many transcriptional targets, including gene responsible for proliferation (e.g. *MYC*), anti-apoptosis (e.g. *BIRC5*), epithelial-mesenchymal transition (e.g. *Snail*), invasion (e.g. *MMPs*), angiogenesis (e.g. *VEGF*), inflammation (e.g. *IL-6*)and stemness (e.g. *SOX2*) (152). β-catenin plays a role in cell-cell adhesion, is a component of adhesion junctions and facilitates the assembly of adhesion junctions (151). HSCs express several different Wnt receptors and various components of Wnt signaling like Wnt3a and Wnt5a promote HSC activation (153), and have been shown to be critical in the onset and progression of fibrosis (149). Thus, activation of the Wnt/β-catenin pathway not only regulates tissue development and regeneration but also affects tumorigenicity and enhances metastatic potential in HCC (147, 154). A growing body of evidence links Wnt/β-catenin to adiposity, body fat distribution and metabolic dysfunction in humans. It can regulate hepatic lipid metabolism and AT function by modulating other regulatory cytokines such as sterol regulatory element-binding protein 1 (SREBP-1), fatty acid synthase (FAS) and the peroxisome proliferator-activated receptor (PPAR) family (155, 156). In addition, Wnt/β-catenin plays a pivotal role in modulating cross-talk between different components of tumour microenvironment (TME), including immune cells, stem cells and non-cellular components of the TME in HCC (157). All of the above support that the Wnt/β-catenin pathway is a potential molecular-targeted therapy in HCC.

### 3.3 JAK/STAT pathway

The Janus protein tyrosine kinase (JAK)/STAT pathway, is a vital downstream mediator for diverse cytokines (IL-6), hormones (leptin) and growth factors (EGF), and is dysregulated in the context of obesity and metabolic disease, including HCC. JAKs and STATs can regulate adipocyte development, such as adipogenesis and transition from preadipocytes to adipocytes, as well as the function of mature adipocytes, and the persistent activated of STAT can lead to deleterious pathological manifestations (158, 159). Accumulating evidence shows that JAK/STAT pathway involves multiple metabolic processes like insulin sensitivity, gluconeogenesis and adiposity (160, 161).

Adipocyte JAK2 and STAT5 deficiency leads to hepatic lipid accumulation, hepatic steatosis, IR and tumorigenesis (160). Hepatic growth hormone (GH) plays an important role in lipid metabolism, systematic glucose metabolism energy supply and cellular regeneration through activating JAK2/STAT5 pathway. Obesity and excess glucose inhibit the secretion of GH, which disrupts GH/JAK2/STAT5 signaling, resulting in excess hepatic lipid accumulation and promoting the process of NAFLD and subsequent HCC (162). STAT3 is closely related to liver injury and plays a pivotal role in the pathogenesis of liver diseases. IL-6 is the most well-described activator of STAT3. The activation of IL-6/JAK/STAT3 signaling in the liver promotes the development of obesity-associated HCC through exacerbating metabolic stress-induced inflammation and immune response (163). Intriguingly, obesity-driving NASH and HCC depend on different STAT signaling pathways (81). In the context of obesity, the oxidative hepatic environment inactivates T cell protein tyrosine phosphatase (TCPTP), a negative regulator of STAT1 and STAT3, and increases STAT1 and STAT3 activity. While the enhanced STAT1 facilitates the recruitment of activated cytotoxic T cells and the consequent NASH and fibrosis. Conversely, STAT3 promotes HCC in obese patients, independent of T cell recruitment, NASH and fibrosis (164). In addition, JAK/STAT signaling controls a diversity of cellular functions, including cell proliferation, stem cell maintenance, differentiation, invasion and metastasis (165).

### 3.4 PI3K/AKT pathway

In obesity, T2DM and NAFLD, hyperinsulinemia and dysregulated insulin signaling occurs when insulin and IGF-1 bind to their respective receptors and activate PI3K/Akt signaling, a key oncogenic pathway for metabolism, cell growth and cell survival (66, 88). In turn, the dysregulated PI3K/Akt pathway further exacerbates the development of obesity, T2DM and subsequent HCC.

AKT regulates lipid metabolism and hepatic lipid content homeostasis. The PI3K/AKT signaling pathway stimulates the gene expression of proteins and transcription factors involved in DNL, including acetyl-CoA carboxylase alpha (ACCα) and sterol regulatory element binding transcription factor 1 (SREBP1) (166). Overexpression of AKT increases glucose uptake, and PI3K/AKT-mediated dysfunction of glucose transport and glycogen synthesis plays an important role in the development of obesity and T2DM (167). AKT2 is a major AKT isoform expressed in insulin-sensitive tissues like liver, its liver-specific deletion inhibits hepatic TG accumulation, further supporting the importance of PI3K/AKT signaling activation in obesity-associated HCC (168). Phosphate and tensin homolog (PTEN), a negative regulator of the PI3K/Akt pathway, suppresses the expression of enzymes involved in hepatic DNL and IR. PTEN is downregulated in the livers of NASH and HCC patients, the deletion of PTEN activates PI3K/AKT, and elevates the levels of SREBP-1c and lipogenic genes, promoting the development of NASH and HCC (88).

### 3.5 MAPK pathway

The family of mitogen-activated protein kinases (MAPKs) mainly includes the stress-responsive MAPKs, c-JNK and p38 MAPK. The associated inflammatory state in obese and insulin-responsive tissues activates stress-responsive p38 MAPKs and JNKs. MAPKs play a prominent role in regulating diversity metabolism processes (169).

JNK is highly activated in NASH-HCC, and the activation of JNK is related to the degree of liver histology activity and promotes the development and carcinogenesis of NASH (69). In obesity and hyperinsulinemia, the increased FFAs, ROS and TNF-α lead to JNK activation in hepatocytes and macrophages, which can increase the production of inflammatory cytokines that can cause inflammation, apoptosis, hepatic IR, liver injury and fibrosis, supporting the metabolic contribution of JNK pathway (66, 170). JNK hyperactivation in macrophages is required for tissue infiltration and pro-inflammatory differentiation, the JNK1 deficiency in macrophages leads to a protective effect against the development of hepatic IR (171). JNK is directly involved in the inhibition of fatty acid oxidation and susceptibility to steatosis through the inhibition of hepatic PPARα and its target genes. In addition, JNK is involved in lipotoxicity and triggers the apoptosis pathway by activating proapoptotic proteins like Bcl-2-like protein 11 (Bcl2-L-11), Bcl2-associated agonist of cell death (BAD) and Bcl-2-like protein 4 (Bcl2-L-4) (40).

Hepatic p38α/β MAPK stimulates hepatic gluconeogenesis by driving the activation of gluconeogenic genes including phosphoenolpyruvate carboxy kinase (PEPCK), glucose-6-phosphatase (G6Pase), and peroxisome proliferator-activated receptor gamma coactivator-1A (PGC-1α) (170). Recent studies demonstrate that activation of p38α MAPK promotes ER, IR and accelerates NASH pathogenesis (169) as well as being elevated in obese patients with NAFLD (172).

### 3.6 AMPK pathway

AMP-activated protein kinase (AMPK) is an intracellular energy sensor that plays a vital role in maintaining energy homeostasis and is involved in diverse biological processes. AMPK activity is increased by nutrient deprivation and reduced in response to inflammation, obesity and NAFLD (52). Loss of AMPK activity exacerbates liver injury and hepatic fibrosis, while increasing AMPK activity has been viewed as a viable therapeutic strategy to improve NAFLD and decrease the risk of NASH, cirrhosis and HCC via three mechanisms: i) suppression of DNL in liver, ii) increased FFA β-oxidation and iii) promotion of mitochondrial function/integrity in AT (88, 173).

In obese humans, ablation of AMPK activity in AT leads to IR and increased liver lipid accumulation (173). In macrophages, AMPK promotes anti-inflammatory phenotypes by inhibiting NF-κB and JNK-mediated pathways, and alleviates the expression of pro-inflammatory genes, such as CCL2 and TNF-α. Activation of AMPK ameliorates liver fibrosis through a variety of mechanisms, including reducing the stimulation of fibrosis, preventing HSCs activation/proliferation/migration and inhibiting the expression of fibrotic genes (174). In addition, AMPK regulates cell proliferation through inhibiting mTOR signaling. Accumulating evidence confirms that hepatic AMPK activity is greatly diminished in NAFLD and NASH, while liver-specific activation of AMPK reduces adipogenesis and completely protects against hepatic steatosis and fibrosis *in vivo* (175).

### 3.7 NF-κB and toll-like receptor pathways

NF-κB and toll-like receptors (TLRs) are key inflammatory pathways associated with obesity-associated HCC (176). Obesity-associated chronic low-grade inflammation is partly mediated by saturated fatty acids stimulating pro-inflammatory pathways in a TLR4-dependent manner in adipocytes and macrophages (40). TLR signaling is able to activate transcription factors (NF-κB and AP-1) and promotes the secretion of inflammatory cytokines (IL-6, IL-1β and TNF-α). These high levels of pro-inflammatory cytokines in hepatocytes cause IR, hepatocytes injury and promote NAFLD, NASH and HCC progression. The elevated IL-1β in KCs, regulated by TLR4, leads to steatosis, inflammation and fibrosis (177). The effect of TLRs on the gut microbiota is an important factor in the relationship between inflammation and obesity. One study shows that mice with TLR5 deficiency have a unique gut microbiota that makes them sensitive to obesity and metabolic syndrome (178).

NF-κB is a transcription factor that plays crucial roles in inflammation, immunity, cell proliferation and the development of liver injury, fibrosis and HCC (179). IKKα/IKKβ complex directly activates NF-κB and is associated with the gene expression downstream of TLRs and cytokines. In the context of obesity, the activation of NF-κB in hepatocytes contributed to IR, increased FFAs, and glucose intolerance (178). In turn, FFA flux can activate NF-κB, via promoting hepatic lipotoxicity, suggesting a potential link between elevated circulating or tissue lipid concentrations and the part of the immune system that mediates inflammation (66). NF-κB has a wide range of functions in different cellular compartments, affecting hepatocyte survival, inflammation in KCs, and survival, inflammation and activation of HSCs (180). For instance, NF-κB participates in activating HSCs and promotes pro-fibrogenic HSC phenotype. NF-κB plays a pivotal role in modulating HSCs survival and promoting the induction and secretion of inflammatory chemokines, including CCL2 and CCL3 (181). On the other hand, NF-KB plays a protective role in the liver and the pronounced inhibition of NF-KB leads to apoptosis of hepatocytes (180). Genetic models lacking major regulators of NF-κB activation such as *Ikkβ*^-/-^ and *Nemo*^-/-^ resulted in a severe embryonic lethality phenotype with significant hepatocyte apoptosis (182).

### 3.8 p53 pathway

The tumor suppressor gene *p53* has emerged as an important regulator of hepatic homeostasis and dysfunction through the integration of cellular stress responses, metabolism and cell cycle regulation, which plays a vital role in the pathogenesis of NAFLD and NASH (154). Under normal circumstances, moderate and temporary p53 activation inhibits the accumulation of liver lipids and inflammation. While exposed to sources of cellular stress such as NASH and overnutrition, the hyper-activation of p53 triggers IR, lipid accumulation, inflammation and oxidative stress in different ways, increasing the risk of HCC (183, 184).

In the context of obesity and hyperglycemia, p53 expression is increased. Elevated p53 level exacerbates the release of pro-inflammatory cytokines and leads to metabolic abnormalities that contribute to the development and progression of HCC (185, 186). For example, in the presence of hyperglycaemia or excessive calorie intake, p53 is activated and leads to systemic IR (187). High p53 levels, whether induced as a response to adiposity or as a trigger for adiposity, may be counterproductive to maintaining AT homeostasis. Recent studies highlight that p53 is essential for regulating the formation of white and brown AT and is also a suppressor of pre-differentiation of adipocytes (187). In AT, the activation of p53 promotes the expression of pro-inflammatory adipokines through NF-κB signaling, leading to hepatic steatosis, IR and diabetes, while inhibition of p53 activity impairs inflammation and attenuates hepatic steatosis (185, 188). p53 is also a major positive regulator of hepatocyte lipid metabolism, and it is involved in lipotoxicity-mediated NASH progression (189). In addition, activation of p53 increases apoptosis of hepatocytes, leading to HSCs activation and the development of liver fibrosis, whereas ablation of p53 completely abolishes this fibrotic phenotype (190).

## 4 Preclinical animal models of NAFLD, NASH and HCC

Human data on liver disease progression is sparse and often limited to a single point in time due to limited access to liver tissue. *In vitro* models do not fully reflect the hepatic and extrahepatic conditions of human NASH. To better understand the pathogenic mechanisms and develop innovative therapies for human obesity-associated HCC, preclinical experimental animal models have been developed to mimic the major features of NAFLD/NASH/HCC, including genetic, metabolic, histologic, as well as proteomic, lipidomic and transcriptomic changes (84). To date, animal models of NAFLD/NASH/HCC can be roughly classified as diet-induced, genetic, toxic or a combination of more than one intervention (**Table 1**).

**Table 1:** Animal models for NAFLD, NASH and HCC.

Model	obesity	IR	NAFLD	NASH	Fibrosis	HCC	Refs
Diet models							
MCD	No	No	Yes	Yes	Yes	No	(191, 192)
CDAA	No	Yes	Yes	Yes	Yes	Low probability	(193)
HFD	Yes	Yes	Yes	Yes	Yes	No	(194)
WD	Yes	Yes	Yes	Yes	Yes	Yes (8-13 months)	(195)
HFD + MCD						Yes	(196)
HFD + CDAA	Yes	Yes	Yes	Yes	Yes	Yes (24-36 weeks)	(84)
HFD + fructose	Yes	Yes	Yes	Yes	Yes	No	(197)
ALIOS	Yes	Yes	Yes	Yes	Yes	Yes (more than 12 months)	(196, 198, 199)
Diet & Toxin models							
STAM	No	No	Yes	Yes	Yes	Yes (16 weeks)	(194, 200)
HFD + DEN	Yes	Yes	Yes	Yes	Yes	Yes (9 months)	(201)
CDAA-HFD + DEN	Yes	Yes	Yes	No	No	Yes (20 weeks)	(84)
WD + CCl4	Yes	Yes	Yes	Yes	Yes	Yes (24 weeks)	(202)
Genetic & Diet models							
*ob/ob* mice + MCD or HFD	Yes	Yes	Yes	Yes	No	No	(199, 203)
*db/db* mice + MCD	Yes	Yes	Yes	Yes	Yes	No	(199, 203)
*foz/foz* mice + WD	Yes	Yes	Yes	Yes	Yes	Yes (56 weeks)	(84, 204)
*Pparα^-/-^* mice + HFD	Yes	No	Yes	Yes	Yes	Yes (24 weeks)	(84)
*Mc4r^-/-^* mice +HFD	Yes	Yes	Yes	Yes	Yes	Yes (48 weeks)	(84, 205)
*Hnf4α^-/-^* mice + HFD	Yes	No	Yes	Yes	Yes	Yes (36 weeks)	(84, 206)
*MUP-uPA* transgenic + HFD	Yes	Yes	Yes	Yes	Yes	Yes (40 weeks)	(207, 208)
Genetic models							
Pten *^-/-^* mice (hepatocyte specific)	No	Yes	Yes	Yes	Yes	Yes (40 weeks)	(84, 196)
Alr*^-/-^* mice	No	No	Yes	Yes	No	Yes (12 months)	(209, 210)
*Aox ^-/-^* mice	No	No	Yes	Yes	Yes	Yes (60 weeks)	(84)
*Mat1a^-/-^* mice	No	No	Yes	Yes	Yes	Yes (72 weeks)	(84)
*Srebp-1c* transgenic	No	Yes	Yes	Yes	Yes	Yes	(207, 211)

### 4.1 Dietary animal models

A diet-induced obesity model, whose macro-nutritional profile is similar to that of obese humans, is the popular NASH mouse model (207). Methionine and choline-deficient (MCD) diet is the most frequently used diet to induce measurable hallmarks of NAFLD and produce the most severe phenotype of NASH in the shortest time. This diet is high in sucrose (40%) and fat (10%), and is deficient in methionine and choline, which are crucial for hepatic β-oxidation and the release of VLDL. In addition, MCD alters glucose metabolism, increases fat accumulation in the liver, and induces significant fibrosis and liver injury (212). However, the MCD model is associated with weight loss, lacking systemic IR, no residual AT and no HCC occurrence (191).

An alternative model uses the Choline-deficient L-amino-defined (CDAA) diet, which is deficient in choline, but proteins in the formula are replaced with an equivalent and corresponding mixture of L-amino acids. Similar to MCD diet, CDAA promotes lipid synthesis, inflammation, steatohepatitis, liver fibrosis and HCC (200). After prolonged CDAA feeding, mice develop obesity, IR and elevated plasma TG and cholesterol (213).

A high-fat diet (HFD) composed of 71% fat, 11% carbohydrates and 18% proteins, can directly increase hepatic FFA accumulation and trigger mitochondrial dysfunction. The HFD model is known to develop IR, inflammation, hepatocyte apoptosis, NASH and fibrosis (194).

Western diet (WD) contains 21.1% fat, 41% sucrose, and 1.25% cholesterol, supplemented with high sugar solution (23.1 g/L d-fructose plus 18.9 g/L d-glucose) in drinking water (202). WD induces obesity, IR and dyslipidemia, activates inflammatory, apoptotic and fibrogenic pathways, and progresses NAFLD, fibrosis, NASH and HCC (212).

Finally, the American lifestyle induced obesity syndrome (ALIOS) diet model is enriched in trans-fats (30% of fat content) and fructose (applied by corn syrup-containing drinking water) (213). In ALIOS model, the hepatic expression of lipid metabolism and insulin signaling genes are increased. In addition, ALIOS induces liver inflammation and bridging fibrosis. Mice in ALIOS exhibit early NASH at 6 months and hepatocellular neoplasms after 12 months (198).

### 4.2 Diet plus toxin animal models

Streptozotocin (STZ) is a naturally occurring chemical that is toxic to insulin-secreting β cells and is often used in preclinical settings to induce type I diabetes (209). It may also directly cause insulin-independent hepatotoxic effects. The combination of STZ with HFD generates a STAM model. In this model, mice are given a low-dose of STZ through intraperitoneal or subcutaneous injection shortly after birth and then feed HFD at 4 weeks of age. This model leads to simple steatosis after 5 weeks, NASH after 7 weeks, followed by fibrosis after 9 weeks, adenomas after 12 weeks and evidence of HCC at approximately 16 weeks (196). The STAM model therefore rapidly induces NASH, however, the mice are lean and have insulin deficiency. Its pathological mechanisms are different from human NASH (200).

Diethylnitrosamine (DEN) is also a chemical to model HCC in mice. DEN induces severe oxidative stress and DNA damage, and promotes lipotoxicity and liver fibrosis. The combination of DEN with HFD or with HFD+CD leads to the occurrence of NASH-associated HCC. In this model, the treated mice exhibit obesity and hepatic steatosis after 8 weeks, IR, lobular inflammation and fibrosis after 12 weeks, and develop into HCC within 20 weeks (200). DEN is a procarcinogen and may be relevant in carcinogenesis, this may represent a substantial difference from its human counterpart (196).

As a hepatotoxin, carbon tetrachloride (CCl4) has been broadly used for inducing liver injury and fibrosis in mice (202). CCl4 triggers oxidative stress and necrotic responses in the liver, leading to liver injury, inflammation and excessive activation and proliferation of HSCs (200). The combination of WD and weekly CCl4 has the advantage of rapid disease progression as mice develop stage III fibrosis at 12 weeks and HCC at 24 weeks (209). More importantly, transcriptome analysis revealed close similarities between the model and human NASH. The CCl4 model can be used to study the progression from simple steatosis to NASH to cirrhosis and HCC (200).

### 4.3 Diet plus genetic animal models

Leptin deficiency (*ob/ob* mice) has been a frequently used model of general metabolism and NAFLD research for a long time (199). *Ob/ob* mice are hyperphagic, inactive and develop severe obesity, hyperlipidemia, hyperglycemia, hyperinsulinemia, and IR, but do not progress to severe liver damage and NASH on a normal diet unless fed with HFD or MCD (214). However, *ob/ob* mice are resistant to liver fibrosis due to leptin requirement (215). Leptin receptor deficiency (*db/db* mice) carries a spontaneous mutation in the *db* gene encoding the leptin receptor, which leads to defective leptin signaling (203). Similar to *ob/ob* mice, *db/db* mice are hyperphagic, obese and IR, and spontaneously develop liver steatosis under normal dietary conditions. *Db/db* mice alone are good models for NAFLD, but not for NASH. However, NASH can be induced when the *db/db* mice are fed with MCD diet. Unlike *ob/ob* mice, *db/db* mice are more susceptible to liver fibrosis (214). In addition, spontaneous mutations in the Alstrom syndrome 1 gene encoding for a protein localized to centrosomes and appetite-sensing neuronal cilia (*foz/foz* mice), *Pparα^-/-^* knockout mice, and melanocortin receptor 4 knockout (*Mc4r^-/-^* mice) lead to overeating, obesity and IR, but do not progress NASH or HCC. HCC is induced in *Mc4r^-/-^* mice and liver-specific *Hnf4α*-deficient mice feeding with HFD within 1 year and 36 weeks, respectively (205, 206). The *foz/foz* mice fed with a WD will present HCC features for more than 56 weeks (204).

Other genetic models like the *Pten* null mice, acyl-coenzyme A oxidase (*Aox*), and methionine adenosyltransferase 1A (*Mat1a*) in global-deficient mice present HCC under normal diet, but show limitations such as no obese phenotype (84).

While the findings from these animal models facilitate our understanding of the pathophysiology of NASH and NAFLD-associated HCC, systematic transcriptome profiling of liver tissues has revealed changes induced by some dietary or genetic models that are not fully mimic the transcriptional profiling of human NASH (106, 216). Due to the complex pathophysiology involved in NAFLD, the ideal animal model representing the complete NAFLD spectrum within a feasible time frame does not exist (217). Researchers should choose the most suitable animal model according to their research objectives, taking into account the comorbidities of NAFLD, the grade of fibrosis and the possible development of HCC.

**Table 2:** Serum markers and score systems for diagnosing NAFLD, NASH and early-stage HCC.

Marker/Score	Parameters	Diagnosis	Refs
Markers			
TG/HDL-C ratio	TG, HDL-C	NAFLD	(227)
BGN	The cutoff value of 189.58 pg/mL of serum BGN with the best sensitivity (93.55%) and specificity (87.18%)	Fibrosis stage of NASH	(228)
CK18	CK18	NAFLD and fibrosis	(229)
Pro-C3	Pro-C3	NASH and different fibrosis stages	(230)
PIIINP	PIIINP	NASH and different fibrosis stages	(34)
Inter-Alpha-Trypsin Inhibitor Heavy Chain 4 (ITIH4)	ITIH4	HCC with NAFLD	(81)
AFP	Ultrasound, AFP	Early stage-HCC with cirrhosis	(221)
Score system			
SteatoTest™	Alanine transaminase (ALT), a2-macroglobulin (A2M), apolipoprotein A-1 (ApoA1), haptoglobin, total bilirubin, gamma-glutamyl transferase (GGT), TC, TG, age gender and BMI	Different stages of steatosis in NAFLD	(218, 231)
NAFLD ridge score	ALT, HDL-C, TG, haemoglobin A1c, white blood cell count, hypertension	NAFLD	(232)
Fatty liver index (FLI)	BMI, TG, waist circumference (WC), and GGT	NAFLD	(233)
Hepatic steatosis index (HSI)	8× (ALT/AST ratio) + BMI (+2, if female; +2, if diabetes mellitus)	NAFLD	(154, 234)
BARD score	BMI, AST/ALT ratio, diabetes, diabetes mellius	NAFLD	(154)
APRI	AST, platelets	NAFLD	(235)
AUROC	Waist circumference, ALT, HbA1c, and HOMA-IR	NAFLD	(154)
Lipid accumulation product (LAP)	WC, TG and gender	Different stages of fibrosis in NAFLD	(225, 236)
NAFLD liver fat score (NAFLD-LFS)	Diabetes, insulin, AST/ALT	Different stages of Fibrosis in NAFLD	(225, 237)
NAFLD fibrosis score (NFS)	Age, hyperglycemia, BMI, platelet count, albumin, AST/ALT ration	Presence of fibrosis in NAFLD	(225)
Index of NASH (ION)	Male: waist-to-hip ratio, TG, ALT and HOMA Female: TG, ALT and HOMA	NASH	(238)
Enhanced Liver Fibrosis (ELF) score	Hyaluronic acid (HA), Tissue inhibitor metalloproteinase 1(TIMP1), and Aminoterminal peptide of procollagen 3 (PIIINP)	Presence of advanced fibrosis in NAFLD	(222)
NASH NIS4	Alpha2 macrogobulin (A2M), Haemooglobin A1c (HbA1c), and Chitinase-3-like protein 1 (CHI3L1)	NASH and advanced fibrosis	(222)
GALAD score	Gender, age, AFP, AFP-L3 and Des-gamma-carboxy-prothrombin (DCP)	Early stage-HCC with NASH	(239)

## 5 Blood-based biomarkers

A key challenge in managing patients with NAFLD is to differentiate NASH from isolated steatosis, as the former carries a high risk of developing cirrhosis and its complications, such as liver failure and HCC. Liver biopsy is the current gold standard for diagnosing NAFLD and NASH, although it is impractical and invasive, may cause life-threatening complications and result in improper diagnosis due to sampling variability (154, 218). Ultrasound has been the main HCC surveillance test for nearly two decades. However, recent data have shown that ultrasound surveillance alone has limitations, including low sensitivity in detecting HCC at an early stage (219). Combining ultrasound with biomarkers, such as alpha-fetoprotein (AFP) may improve the accuracy of early HCC detection (220, 221). Thus, more biomarkers are needed to diagnose NAFLD, NASH with advanced fibrosis and early-stage HCC, which is critically essential for selecting appropriate treatment (81, 222).

During the last decades, diverse non-invasive blood testing has been developed, plasma biomarkers (e.g. high-density lipoprotein cholesterol (HDL-C), biglycan (BGN), cytokeratin 18 (CK18), pro collagen III (Pro-C3) and plasma N-terminal propeptide of type III procollagen (PIIINP)) are commonly used to reflect specific and complex molecular mechanisms underlying the pathogenesis and progression of NAFLD and NASH (154, 223). Currently, AFP is the only biomarker that has completed all phases of biomarker evaluation and has sufficient evidence to be used in clinical HCC detection when combined with ultrasound (224). Serum inter-alpha-trypsin inhibitor heavy chain 4 (ITIH4) is another potential biomarker for NAFLD progression and HCC development (81). Because the pathogenesis of NAFLD and NASH is complex and may involve multiple biological aberrations, it is unlikely that a single biomarker can differentiate simple steatosis from NASH, and identify advanced fibrosis (225). Therefore, composite score systems (e.g. SteatoTest™, Fatty liver index (FLI), BARD score) include at least two or more variables to increase the robustness of the non-invasive predictive model (223). They are certainly clinically useful and avoid liver biopsy in many cases (226). It is worth noting that the GALAD score based on gender, age, AFP, AFP isoform L3 (AFP-L3), and des-gamma-carboxy prothrombin (DCP) has been approved to be used for the early diagnosis of HCC with NASH (81). Here, the serum markers and score systems for NAFLD, NASH and HCC are summarized in **Table 2**.

## 6 Potential therapeutic targets of obesity-associated HCC

### 6.1 Leptin

Leptin, a predominant adipokine secreted mainly by AT, is increased in obese populations and patients with liver disease and is related to NAFLD progression (240). Leptin is central to the obesity-cancer link since it is produced in proportion to fat mass. Leptin is effective in inducing HCC cells mitosis, growth and motility by activating JAK/STAT, PI3K/Akt and ERK signaling pathways. These pathways upregulate cyclin D1 expression, promoting the proliferation of hepatocytes and HCC cells (241–243). Leptin has a pro-inflammatory effect and a high level of leptin causes other inflammatory cells to stimulate the differentiation of monocytes into macrophages, favoring the chronic inflammatory state associated with obesity (244). Leptin also contributes to hepatic fibrogenesis via TGF-β and activating HSCs (245). In addition, high levels of leptin promote angiogenesis through upregulating VEGF. In obese individuals, higher levels of leptin increase the risk of HCC recurrence after curative therapy (246).

### 6.2 Adiponectin

Adiponectin is the most abundant and adipose-specific adipokine secreted by adipocytes, whose reduction plays a central role in obesity-associated HCC. Its level paradoxically increased with the decreasing fat mass (247), and both serum and hepatic levels of adiponectin are decreased in NASH patients (248, 249). It exhibits an anti-inflammatory effect through inhibiting the secretion and action of TNF-α, IL-6 and other proinflammatory cytokines, blocking the activation of NF-κB (250). Adiponectin also displays anti-lipotoxic effects, it is able to promote FFA β-oxidation, prevent lipid accumulation in adipose and hepatic tissues, and regulate glucose homeostasis and hepatic insulin sensitivity (251, 252). Adiponectin exerts an inhibition in the proliferation of adipocyte cells, endothelial cells and tumor cells by activating AMPK and regulating c-JNK/caspase 3 pathways (46). In addition, adiponectin also possesses antiangiogenic properties by decreasing the expression of VEGF. A low level of adiponectin is related to obesity-associated IR and carcinogenesis (253). Hence, it is a novel therapeutic target for obesity-associated HCC (254).

### 6.3 Peroxisome proliferator-activated receptors

Peroxisome proliferator-activated receptors (PPARs) regulate lipid and glucose metabolism and play a key role in hepatic energy homeostasis and the regulation of adipogenesis (52, 154). PPARα negatively regulates hepatic lipid uptake by regulating FFAs transport, esterifying FFA and increasing mitochondrial FFA oxidation. Activation of PPARα inhibits NF-κB-induced inflammatory genes and reduces the level of acute inflammation response genes (255). Therefore, its abnormalities may cause hepatic steatosis, steatohepatitis, fibrosis, and HCC (52). In addition, PPARα enhances the expression of FGF21 and glutamate transporter 1 (GLT1), which improve systemic insulin sensitivity and lipid turnover (256). Activation of PPARβ/δ protects against dyslipidemia, IR, obesity and NAFLD. PPARβ/δ promotes hepatic glucose catabolism and increases HDL cholesterol and shows a strong TAG- decreasing effect. Similar to PPARα, PPARβ/δ has anti-inflammatory effects in the liver by inhibiting NF-κB activity (256). PPARγ is highly expressed in AT and macrophages and plays a key role in adipogenesis, lipid metabolism, insulin sensitivity and immune regulation (257). PPARγ prevents the increased flow of FFAs and adipokines from AT to other organs, especially to the liver (258). The PPARγ activator, rosiglitazone approved by the FDA for the treatment of T2DM, showed effects against steatosis, hepatocellular inflammation, ballooning degeneration and fibrosis (154).

Elafibranor, a PPARα and PPARβ/δ agonist, improves serum lipid profile and IR and improves NASH without worsening fibrosis in Phase II clinical trials (259). Saroglitazar, a major PPARα and moderate PPARγ agonist, has also been reported to improve liver enzymes, liver fat content, IR and atherosclerotic dyslipidaemia in participants with NAFLD/NASH (260). Lanifibranor, a pan-PPAR agonist, ameliorates diet-induced NASH through upregulation of β-oxidation and FA desaturation (47).

### 6.4 Farnesoid X receptor

Farnesoid X receptor (FXR), a bile acids-activated nuclear receptor, is highly expressed in intestine, liver and kidneys. FXR is responsible for hepatic glucose and lipid metabolism, carbohydrate metabolism, inflammation, bile acid production, as well as lipoprotein composition, immune responses and insulin signaling (106, 114). More importantly, the excessive activation of FXR triggers a steady release of FGF19, which is an atypical hormonal regulator of metabolism and bile acid homeostasis that has been associated with improvements in NASH (38). In preclinical studies, FXR activation has been shown to attenuate hepatic steatosis, reduce lipotoxicity and inflammation, increase insulin sensitivity, and exhibit direct anti-inflammatory and antifibrotic effects, suggesting that modulation of FXR has beneficial effects on obesity-related liver diseases (207).

Obeticholic acid (OCA) is one of the FXR agonists that has reached Phase III clinical trial. OCA exhibits excellent effects in NASH patients, it improves hepatic inflammation, fibrosis and hepatic damage (261). Cilofexor (GS-9674), a non-steroidal agonist of FXR with anti-inflammatory and anti-fibrotic effects, has completed a Phase II clinical trial. Cilofexor significantly improved hepatic steatosis and reduced serum γ-glutamyl transferase, C4 and primary bile acids in NASH patients, but did not improve liver fibrosis and stiffness (154). Other FXR agonists include tropifexor (LJN452), TERN-101, EDP-305, EYP001a and LMB763 (47, 52).

### 6.5 Stearoyl-CoA desaturase 1

Stearoyl-CoA desaturase 1 (SCD1), a key enzyme in DNL and fatty acid metabolism, controls a rate-limiting step in mono-unsaturated fatty acid synthesis and has been considered a promising target for NAFLD treatment (262). Obesity and hepatic steatosis are known to strongly induce SCD1 expression, whereas rodents that are specifically deficient in SCD1 in the liver are protected from developing hepatic steatosis by reducing lipid synthesis and increasing FFA β-oxidation and insulin sensitivity (38). Inhibition of SCD1 produces a number of beneficial effects, including reducing liver fat, preventing IR and obesity. Aramchol, an oral SCD1 inhibitor targeting liver, decreased the liver fat content and improved liver histology in a Phase II clinical trial without exhibiting toxicity. Aramchol is being further evaluated as a drug candidate for the treatment of NAFLD in an ongoing Phase III trial (114). In addition, a number of synthetic SCD1 inhibitors, including CVT-12012, GSK1940029, MF-438, MK-8245 and SW203668, are being evaluated for efficacy in preclinical and clinical studies (52).

### 6.6 Acetyl-CoA carboxylase

It is known that increased hepatic DNL contributes to NASH, while the rate-limiting step in DNL is catalyzed by Acetyl-CoA carboxylase (ACC), suggesting inhibition of ACC represents an attractive approach for the treatment of NASH (263). ACC has two major isoforms, ACC1 and ACC2. ACC1 is localized on the cell membrane and is expressed in liver and AT, whereas ACC2 is expressed on the mitochondrial surface of oxidative tissues, such as liver, heart and skeletal muscle (47). Inhibition of ACC1 and ACC2 reduces DNL and increases FA β-oxidation. Firsocostat (GS-0976) is a hepatic ACC1 and ACC2 inhibitor that reduces steatosis, inhibits DNL and reduces serum fibrosis markers in non-cirrhotic NASH patients in a Phase II trial (264).

### 6.7 Fatty acid synthase

Fatty acid synthase (FASN) catalyzes the conversion of malonyl CoA and acetyl CoA to the saturated C16 fatty acid palmitate, which plays a key role in DNL, making this multi-catalytic protein an attractive therapeutic target for obesity, and associated liver diseases (265). FASN inhibition decreases TG content, consistent with direct anti-steatotic activity. Denifanstat (TVB-2640), an inhibitor of FASN, is in a Phase II clinical trial for NASH and is being used in the primary human liver microtissue (LMT) study (266).

### 6.8 Glucagon-like peptide-1

Glucagon-like peptide-1 (GLP-1) is an endogenous hormone, secreted by intestinal endocrine L-cells that regulates blood glucose levels. GLP-1 enhances the release of insulin, induces fatty acid oxidation in hepatocytes, inhibits glucagon secretion and reduces food intake by binding to the GLP-1 receptor (GLP-1R). Inactivation of GLP-1 leads to glucose intolerance, T2DM and hepatic steatosis (52), suggesting GLP-1 is a potential medication for NAFLD. GLP-1 activity is significantly decreased due to the actions of a protease DPP4, which cleaves GLP-1 and has a higher level in NASH patients (38, 52). Exenatide, the first GLP-1 analogue, is resistant to DPP4 and its secondary and tertiary structures, with a much longer half-life and hypoglycemic effect (52). It is able to decrease serum ALT levels, and improve hepatic fat and fibrosis (267). Liraglutide, another GLP-1 agonist requiring daily injection, results in increased insulin sensitivity, decreased DNL, reduced BMI and suppression of lipolysis in patients with NASH (268). Liraglutide is safe, well tolerated and leads to histological resolution of NASH (269). In addition, the DPP4 inhibitors, sitagliptin and evogliptin can prolong the half-life of GLP-1 and improve NASH (52).

### 6.9 Sodium-Glucose Cotransporter-2

Sodium-Glucose Cotransporter-2 (SGLT2) is expressed almost exclusively in the kidney, where more than 90% of the glucose filtered by the glomerulus is reabsorbed. SGLT-2 is profoundly involved in the regulation of inflammatory responses, fibrogenesis and many intracellular signaling pathways (270). SGLT-2 inhibitors increase glucagon levels, reduce renal reabsorption of glucose, and promote the loss of calories in the urine, with subsequent weight loss (268). Many studies have indicated that SGLT-2 inhibitors improve liver function and liver fibrosis, suggesting SGLT-2 inhibitors hold promise for treating NASH.

In patients with T2DM and NAFLD, inhibition of SGLT2 by dapagliflozin attenuates liver fibrosis and steatosis, and decreases the serum level of DDP4. The safety and efficacy of dapagliflozin in NASH patients is being assessed in a Phase III clinical trial (271). Other SGLT2 inhibitors currently in use, include canagliflozin, ipragliflozin, ertugliflozin and empagliflozin, which have multiple functions in the treatment of NAFLD and T2DM by preventing DNL, hepatic inflammation and apoptosis, and increasing fatty acid oxidation (154).

### 6.10 Thyroid Hormone Receptor-β

Thyroid Hormone (TH) is involved in myriad essential cellular and organismal functions like hepatic TG and cholesterol metabolism by binding to two Thyroid Hormone Receptor (THR), THR-α and THR-β (38). THR-β is highly expressed in hepatocytes and is responsible for regulating metabolic pathways in the liver that are often compromised in NAFLD. The THR-β level in liver is reduced in patients with NASH (272). Selective engagement of the THR-β subtype in the liver has emerged as a potential approach for the treatment of NASH. Activation of THR-β is able to decrease TG and cholesterol levels, improve insulin sensitivity, reduce apoptosis, increase fat oxidation and promote liver regeneration (262). Resmetirom is a selective THR-β agonist that likely reduces liver fat, enhances fatty acid catabolism and alleviates hepatic steatosis and dyslipidemia. Currently, resmetirom is being evaluated for safety and efficacy in patients with NASH and fibrosis in a Phase III clinical trial (261).

### 6.11 Fibroblast growth factors 19 and 21

Circulating FGF21 is derived from the liver and is also expressed in several other tissues, such as the pancreas, muscle and adipose (258). FGF21 has been shown to play a vital role in regulating organ metabolism and systematic energy homeostasis, especially hepatic lipid metabolism. FGF21 enhances lipid oxidation, inhibits lipolysis in AT, suppresses DNL in the liver, and improves insulin sensitivity by inhibiting mTOR (114, 273). Deficiency of FGF21 favors the development of steatosis, inflammation, hepatocyte injury and fibrosis in the liver, while administration of FGF21 analogues improves NASH by attenuating these processes (274). Pegbelfermin (BMS-986036), a recombinant FGF21 analogue, has been used in clinical trials for patients with NASH and stage 3 fibrosis. Subcutaneous treatment of pegbelfermin reduces liver fat, and improves biomarkers of metabolic function and fibrosis (261). Efruxifermin, an FC-FGF21 fusion protein, is able to improve NAFLD activity score (NAS) and fibrosis, and reduce body weight and liver fat content in clinical Phase II trials (261).

FGF19 is a gastrointestinal hormone that regulates bile acid synthesis, glucose metabolism and hepatic fatty acid oxidation and is a known downstream regulator of FXR (258). Circulating FGF19 concentration is decreased in NASH patients, but FGF19 can also stimulate tumour progression through activating STAT3 pathway (275). NGM282, a humanized FGF19 analogue, acts in the same way as FXR agonists (47). In clinical trials, NGM282 is able to reduce AST and ALT levels, and improve liver fat content, fibrosis and liver transaminases (47, 52, 258).

### 6.12 The C-C chemokine receptors 2 and 5

The C-C chemokine receptors 2 and 5 (CCR2 and CCR5) and their respective ligands (CCL2 and CLL3-5) are implicated in the pathogenesis of liver inflammation, immune cell infiltration and fibrosis, leading to the development of NAFLD and NASH (114). Cenicriviroc is a novel dual CCR2 and CCR5 antagonist currently in clinical development for the treatment of liver fibrosis in NASH patients. It blocks overactive inflammatory signals and disrupts signals that activate stellate cells, thus targeting the onset of inflammation and fibrosis (264). In Phase II clinical trial, cenicriviroc exhibited a significant improvement in fibrosis of NASH patients. However, based on the results of the Phase III AURORA trial, it was terminated early due to lack of efficacy (258).

### 6.13 Galectin-3

Galectin-3 is a β-galactoside binding protein mainly secreted by macrophages. Its expression is increased in NASH, and it is associated with the severity of fibrosis and inflammatory responses (276). Galectin-3 also modulates diverse physiologic and pathologic processes, including cell apoptosis, adhesion, migration and angiogenesis (277). The ablation of Galectin-3 decreases hepatic advanced lipoxidation endproduct (ALE) accumulation and improves inflammation, hepatocyte injury and fibrosis (278). GR-MD-02 is an inhibitor of Galectin-3, which has shown promising results for NASH patients with fibrosis in clinical trials (52).

### 6.14 Apoptosis signal-regulated kinase 1

Apoptosis signal-regulated kinase 1 (ASK1)1 plays a pivotal role in regulating hepatocyte injury, inflammation, apoptosis and fibrosis in NASH through c-JNK signaling (279). Selonsertib is a first-in-class inhibitor of ASK1 that has been shown to prevent inflammation, fibrosis, excessive apoptosis and progression to cirrhosis in a Phase II clinical trial in patients with NASH and stage 2-3 fibrosis (114). However, the Phase III clinical trial was terminated since it failed to reach primary and secondary endpoints (258).

## 7 Clinical trials

Currently, there are no drugs that have been approved for NAFLD/NASH treatment, and treating this disease remains a major unmet clinical need (280). However, within the past decade, a number of studies have been investigating new drugs for NASH, improving developments in this area. Consequently, many drugs are now undergoing various stages of clinical trials in NAFLD/NASH patients. Based on the pathophysiologic classification of NASH, these drugs include insulin sensitizers, anti-DNL drugs, lipid-lowering drugs and anti-fibrosis drugs. In addition, other clinical trials for anti-inflammation and anti-apoptosis agents are also ongoing. Below are the current pharmaco-therapeutic options that are in clinical trials (**Table 3**).

**Table 3:** Ongoing clinical trials for NAFLD/NASH patients.

Medication	Primary mechanism	Inclusion criteria	Clinical trial number	Trial phase
Insulin sensitizer				
Metformin	Insulin sensitizer	NAFLD	NCT01084486	Phase II
Pioglitazone	PPARγ agonist	NASH, NAFLD, T2DM	NCT00994682	Phase IV (completed)
MSDC-0602K	Mitochondrial pyruvate carrier (MPC) inhibitor	NAFLD, NASH	NCT02784444	Phase II
Inhibition of DNL and lipotoxicity				
Aramchol	SCD1 inhibitor	NASH, NAFLD	NCT04104321	Phase III
Firsocostat (GS-0976)	ACC inhibitor	NASH	NCT02856555	Phase II
TVB-2640	FASN inhibitor	NAFLD	NCT04906421	Phase II
Elafibranor	PPARα/β agonist	NASH with fibrosis	NCT02704403	Phase III (terminated due to failure of the predefined primary surrogate efficacy endpoint)
Seladelpar (MBX-8025)	PPARδ agonist	NASH	NCT03551522	Phase II (terminated due to unexpected histological findings)
Saroglitazar	PPARα/γ agonist	NAFLD	NCT03617263	Phase II
Lanifibranor (IVA337)	Pan PPAR agonist	NASH	NCT03008070	Phase II
Obeticholic Acid (OCA)	FXR agonist	NASH	NCT02548351	Phase III
Cilofexor (GS-9674)	FXR agonist	NASH	NCT02854605	Phase II
Tropifexor (LJN452)	FXR agonist	NASH	NCT02855164	Phase II
TERN-101	FXR agonist	NASH	NCT04328077	Phase II
EDP-305	FXR agonist	NASH	NCT03421431	Phase II
EYP001a	FXR agonist	NASH	NCT03812029	Phase II
LMB763	FXR agonist	NASH	NCT02913105	Phase II
Exenatide	GLP-1 agonist	NAFLD	NCT01208649	Phase IV
Liraglutide	GLP-1 agonist	NASH, NAFLD	NCT02654665	Phase III
Semaglutide	GLP-1 agonist	NASH	NCT04822181	Phase III
Dulaglutide	GLP-1 receptor agonist	T2DM, NASH	NCT03648554	Phase IV
Tirzepatide	Dual GIP and GLP-1 receptor agonist	NASH	NCT04166773	Phase II
BI456906	Dual GIP and GLP-1 receptor agonist	NASH	NCT04771273	Phase II
Sitagliptin	DPP4 inhibitor	NAFLD	NCT01963845	Phase II
Evogliptin	DPP4 inhibitor	NAFLD, T2DM	NCT03910361	Phase IV
LIK066	SGLT1/2 inhibitor	Obese patients with NASH	NCT03205150	Phase II
Empagliflozin	SGLT2 inhibitor	NAFLD, T2DM	NCT02964715	Phase IV
Ipragliflozin	SGLT2 inhibitor	NAFLD, T2DM	NCT02875821	Phase IV
Dapagliflozin	SGLT2 inhibitor	NASH	NCT03723252	Phase III
Canagliflozin	SGLT2 inhibitor	NAFLD with T2DM	NCT05513729	Phase I
Ertugliflozin	SGLT2 inhibitor	NAFLD/NASH with liver fat, liver fibrosis, T2DM	NCT05644717	Phase IV
Resmetirom (MGL-3196)	THR-β Agonist	NASH	NCT03900429	Phase III
VK2809	THR-β Agonist	Hyperlipidemia, NAFLD	NCT02927184	Phase II
NGM282	FGF19 agonist	NASH	NCT03912532	Phase II
Oltipraz	Liver X receptor alpha (LXR-α)	NAFLD	NCT04142749	Phase III
Gemcabene	APOC3 inhibitor	NAFLD	NCT03436420	Phase II (terminated due to lack of efficacy and safety concerns)
Anti-inflammation				
JKB-121	TLR-4 antagonist	NASH	NCT02442687	Phase II
Cenicriviroc	CCR2/5 antagonist	NASH	NCT03028740	Phase III (terminated due to lack of efficacy)
BI 1467335	Amine oxidase copper containing 3 (AOC3) inhibitor	NAFLD	NCT03166735	Phase II
Namodenoson	Adenosine receptor agonist	NASH	NCT04697810	Phase II
IMM-124E	Anti-LPS	NASH	NCT02316717	Phase II
Solithromycin	Anti-LPS	NASH	NCT02510599	Phase II
Anti-fibrosis				
Pegbelfermin	FGF21 agonist	NASH with stage 3 fibrosis	NCT03486899	Phase II
Pegbelfermin	FGF21 agonist	NASH with cirrhosis, NAFLD, Liver fibrosis	NCT03486912	Phase II (completed)
Efruxifermin	FGF21 agonist	NASH	NCT03976401	Phase II
MK-3655	Monoclonal antibody agonist of the b-Klotho/FGFR1c receptor complex	NASH	NCT04583423	Phase II
Belapectin (GR-MD-02)	Galectin-3 inhibitor	NASH	NCT04365868	Phase III
Losartan	Angiotensin II receptor blocker	NASH	NCT01051219	Phase III
Simtuzumab (SIM)	LOXL2 monoclonal antibody	NASH	NCT02466516	Phase II
ND-L02-s0201 (BMS-986263)	HSP47 siRNA	NASH	NCT04267393	Phase II
MT-3995	Mineralocorticoid receptor antagonist	NASH	NCT02923154	Phase II
AZD2693	Patatin like phospholipase domain containing 3 (PNPLA3) inhibitor	NASH	NCT04483947	Phase I
CC-90001	JNK inhibitor	NASH with Stage 2, Stage 3 liver fibrosis	NCT04048876	Phase II (terminated due to changes in business objectives)
Anti-apoptosis				
Selonsertib	ASK1 inhibitor	NASH	NCT03053063	Phase III (terminated due to lack of efficacy)
Emricasan (ENCORE-NF)	Caspase Inhibitor	NASH, Fibrosis, liver disease	NCT02686762	Phase II
Antioxidants				
Vitamin E	Antioxidants	NAFLD, NASH	NCT04801849	Phase II
Pentoxifylline	Phosphodiesterase inhibitor	NASH	NCT05284448	Phase III

## 8 Conclusion

Obesity is a highly prevalent and recurrent disease that increases the risk of HCC by exacerbating the onset and progression of NAFLD and NASH. Extensive studies have focused on understanding the pathophysiology of NAFLD/NASH and obesity-associated HCC, including the molecular mechanisms and related signaling pathways. A diversity of preclinical experimental animal models have been developed to facilitate *in vivo* research, each with advantages and limitations depending on the research hypothesis. More recently non-invasive technologies such as serum biomarkers have been developed for the early diagnosis of NAFLD/NASH. Numerous candidate drugs have exhibited efficacy in fibrosis, inflammation and steatosis in clinical trials. These findings may open up novel approaches to treatment. There remains an unmet need for reliable biomarkers and non-invasive tools to accurately stage the progression of NAFLD/NASH and to validate the safety and efficacy of potential therapies in clinical trials. Furthermore, a personalized medicine approach will be needed to tailor the right therapeutic approach to optimize the treatments in individual patients for obesity-related liver disease.

## Author contributions

YC and SA discussed the content, YC wrote the first draft of the manuscript. All the listed authors reviewed and revised the text. All authors contributed to the article and approved the submitted version.
